# Can We Exclude the Diagnosis of Non-ST Segment Myocardial Infarction on the Basis of a Single Troponin I and a Symptom Duration ≥8 Hours?

**DOI:** 10.5402/2011/364728

**Published:** 2011-04-12

**Authors:** Jeremy S. Lynn, Amandeep Singh, Eric R. Snoey

**Affiliations:** Department of Emergency Medicine, Alameda County Medical Center, Highland Hospital, Oakland, CA 94602-1018, USA

## Abstract

*Background*. The use of a single troponin measurement to exclude the diagnosis of non-ST segment myocardial infarction (NSTEMI) in patients that present with ischemic symptom duration ≥8 hours is sometimes used in the Emergency Department. *Study Objective*. To describe the characteristics of patients with initial nondiagnostic troponin values who develop a positive troponin while in the Emergency Department and to evaluate whether NSTEMI can be excluded using symptom duration ≥8 hours and initial troponin I. *Methods*. Retrospective chart review of patients evaluated for NSTEMI in the Emergency Department. *Results*. 4,510 patients had at least two troponin I values obtained during the two-year study period. 115 (2.5%) of these patients had an initially nondiagnostic (<0.6 ng/mL) and subsequent positive (≥0.6 ng/mL) troponin I result. Twenty-five (22%) of the 115 had duration of symptoms ≥8 hours. Of these 25 patients, 18 had an intermediate first troponin value (i.e., >0.06 ng/mL, but <0.6 ng/mL). Only two of the remaining seven patients had a final primary diagnosis of NSTEMI. *Conclusion*. The use of a negative initial troponin I together with a symptom onset of ≥8 hours defines a population with a very low incidence of a hospital diagnosis of NSTEMI.

## 1. Introduction

### 1.1. Background and Importance

Troponin has become the preferred cardiac biomarker with respect to the diagnosis of acute myocardial infarction (AMI) [1–3]. This serum protein of cardiac cell necrosis appears 4 to 12 hours after symptom onset and remains abnormal for 4 to 10 days [4, 5]. To avoid missing early myocardial damage in patients with normal or nondiagnostic electrocardiograms, physicians routinely employ the use of serial troponin measurements initially on arrival to the emergency department (ED) and subsequently at a time frame of 6 to 12 hours after the onset of ischemic symptoms. The precise timing of serum marker measurement, however, must take into account the uncertainties often present with the exact timing of onset of ischemic symptoms and the sensitivity, precision, and institutional norms of the assay being utilized. A strategy of measuring cardiac markers within 6 hours of symptom onset with repeat measurements occurring 8 to 12 hours after symptom onset is recommended by the American College of Cardiology/American Heart Association (ACC/AHA) and is thought to approach 100% sensitivity for excluding the diagnosis of AMI [3]. 

The American College of Emergency Physicians (ACEP) has published a clinical policy that supports the use of a single negative TnI obtained 8 to 12 hours after symptom onset for the exclusion of non-ST-segment elevation myocardial infarction (NSTEMI). There are, however, relatively few published reports substantiating the safety of this approach. Furthermore, a review of the use of troponin T in this setting was unable to confirm the safety of this approach [6]. Additionally, there has been no published research defining the population of patients who present with an initial normal TnI who then develop an elevated value on subsequent testing. As such, little is known about the demographic, historical, physical exam, electrocardiographic, and laboratory features of these patients.

### 1.2. Goals of This Investigation

We sought to define the frequency and clinical characteristics of ED patients with an initial nondiagnostic, but subsequent positive TnI. In addition, we sought to determine what percentage of the aforementioned patients presented with a symptom duration greater than 8 hours. These patients reflect a population that the ACEP guidelines for the diagnosis of NSTEMI would have failed to recognize.

## 2. Methods

### 2.1. Study Design and Setting

All data for this study was obtained through a systematic review of the electronic and paper medical record (when available) for patients evaluated in the emergency department of a single urban county teaching hospital with an annual census of approximately 80,000 visits. Two authors (J. S. Lynn and A. Singh) conducted the data abstraction using a standardized data abstraction worksheet. Approval for this study was granted by the institutional review board at our institution, prior to the commencement of data acquisition.

### 2.2. Selection of Participants

Patients were included in this study if they presented to the emergency department (ED) between January 2004 and December 2005 and fulfilled all of the following criteria: (1) had at least two TnI laboratory values obtained, (2) the first TnI value was ≤0.6 ng/mL, and (3) any subsequent troponin I value in the ED was >0.6 ng/mL. There were no exclusion criteria. A laboratory database of all TnI results obtained during the study period was used to identify participants.

### 2.3. Troponin Assays

From January 2004 through September 2005 the Stratus stat-fluorometric analyzer (Dade-Behring) was used to measure troponin values. The analytical lower threshold for detection for this method was 0.03 ng/mL, the laboratory reference range extended to 0.06 ng/mL, and the threshold for suspecting acute myocardial infarction was ≥0.6 ng/mL [7]. After September 2005, TnI values were determined with the Access 2 (Beckman-Coulter). The analytical lower detection threshold and interpretive ranges remained the same. TnI measurements that were <0.6 ng/mL were labeled as “negative” for AMI, whereas those ≥0.6 ng/mL were considered “positive” for AMI. Values between the lower threshold for detection (i.e., ≥0.06 ng/mL) but less than the diagnostic cutoff for AMI (i.e., <0.6 ng/mL) were considered “intermediate”.

### 2.4. Data Collection and Processing

Data was collected directly onto a standardized secure computerized database. Source documents included all parts of the ED electronic medical record, including triage notes, nursing notes, any physician notes, and laboratory data. In addition, the paper record was reviewed for each subject including the ED physician's history and physical (H&P), admitting resident's H&P, consulting specialist's H&P, and both the written and dictated discharge summary (when available).

Discrepancies among the medical records for a given subject were reconciled according to the following predefined rules. If one or more source listed a given condition in the patient's past medical history, it was recorded as present; otherwise, it was recorded as absent. When determining the duration of symptoms, the most specific time of onset recorded by any provider was used (i.e., 9 AM instead of “this morning”). If two sources had equal specificity, then the later time was recorded. When nonspecific times of the day were all that was available, preassigned times were used as follows—“last night” = 8 PM, “this morning” = 8 AM, or “this afternoon” = 2 PM. 

The duration of symptoms was defined as the duration of time between the onset of symptoms for which the patient presented to the ED and arrival in the ED. For example, if the patient had worsening chest pain for 2 days, and eventually came to the ED, the duration of symptoms was defined as 2 days. However, if a patient had shortness of breath for one week and developed chest pain 3 h prior to arrival, the duration of symptoms was recorded as 3 h.

ECGs were recorded as either diagnostic or non-diagnostic of ischemia. ECGs were considered diagnostic of ischemia if they were recorded by the treating physicians as having any one of the following, not known to be old: >0.2 mV ST depression in 2 or more contiguous leads, >0.1 mV ST elevation in 2 or more contiguous leads, or a left bundle branch block.

### 2.5. Data Analysis

Data was analyzed using descriptive statistical analysis.

## 3. Results

**Figure 1:**
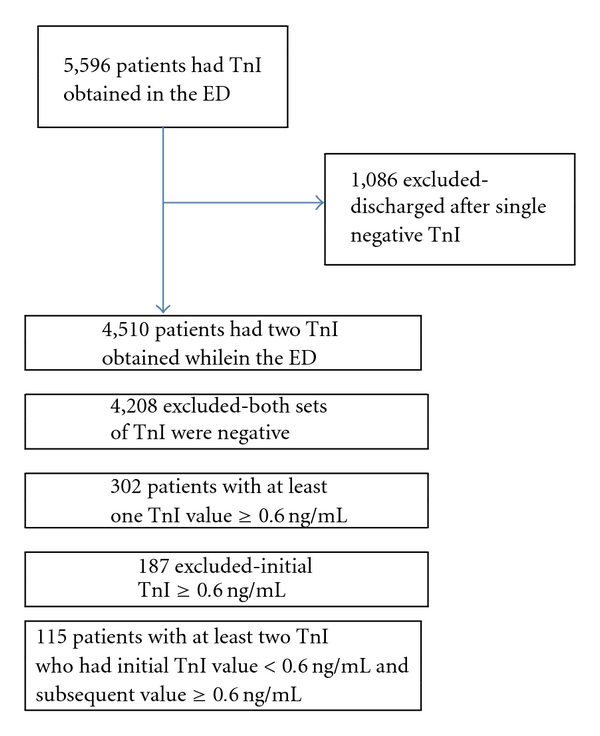


Five thousand five hundred and ninety-six patients presenting to the ED during the study period that had at least one TnI obtained. One thousand and eighty-six patients did not have a second TnI obtained and were therefore excluded. Of the remaining 4,510 patients, 4,208 (93.3%) did not have any TnI levels greater than 0.6 ng/mL. This left a total of 302 (6.7%) patients that had at least one TnI value ≥0.6 ng/mL. Of these 302 patients, 187 (61.9%) had an initial troponin I value that was ≥0.6 ng/mL and were therefore excluded from analysis. The remaining 115 patients were the group of patients who had at least two TnI values obtained, with the first value <0.6 ng/mL and at least one subsequent value ≥0.6 ng/mL. These patients comprised the study population (Figure 1).

Ninety (78.2%) patients had their positive troponin drawn within 8 hours of the first sample. Of the remaining 25 patients that required >8 hours, 11 were not positive until three or more TnI values were obtained.

The characteristics of the study population including age, gender, race, and significant past medical history are listed in Table 1. Traditional cardiac risk factors—hypertension, diabetes, active smoking, hyperlipidemia, or family history of early MI were present in 86.1% of patients with 26.1% having 3 or more concomitant risk factors. If cocaine use, prior stroke, or prior MI were considered in addition to the traditional cardiac risk factors, the percentage of study patients with at least one cardiac risk factor increased to 93%.

Table 2 summarizes the characteristics of the study population upon arrival to the ED. In our institution, patients are activated as a “medical code” if they are thought to require immediate physician and nursing interventions for an unstable medical condition. Forty-seven (40.9%) of the study patients were activated as a medical code. Nine (7.8%) patients were brought in to the hospital as trauma activations. Symptoms were considered “ongoing” if the patient was symptomatic at the time of arrival to the ED. All but 6 patients had ongoing symptoms upon arrival to the ED. These 6 patients all presented after a fall or syncope. The majority of patients (53.0%) had less than 1 hour of symptoms upon arrival to the ED. Ninety-one (79.1%) patients had less than 8 hours of symptoms. That leaves 25 (21.7%) patients who presented with an initial negative but subsequent positive TnI (≥0.6 ng/mL) and had ≥8 hours of symptoms upon arrival to the ED. 

Selected details regarding the hospital course of these 25 patients are presented in Tables 3 and 4. All 25 had ongoing symptoms upon arrival to the ED. Sixteen of the 25 patients had a final primary diagnosis other than acute coronary syndrome. Only one of the patients listed on Table 4 died during the hospital admission—a 73-year-old woman who suffered a massive pulmonary embolism. 

Although 0.6 ng/mL is considered the “cutoff” for the diagnosis of acute MI, the 99th percentile of the upper limit of the TnI reference range for a healthy population is 0.06 ng/mL—an order of magnitude lower than the cutoff for AMI. Forty-eight (41.7%) patients of the 115 in the study had an initial troponin that was <0.06 ng/mL. Of those 48 patients with a true negative first troponin, only 7 patients had ≥8 hours of symptoms at the time of presentation. 

Of all 4,510 patients that had more than one TnI value obtained, only 7 (0.16%) had ≥8 h of symptoms, an initial troponin <0.06 ng/mL and any subsequent troponin ≥0.6 ng/mL. Two of these 7 patients had an ultimate primary final diagnosis of acute coronary syndrome.

## 4. Discussion

In 2006, the ACEP Clinical Policies Subcommittee published guidelines for the evaluation and management of patients with potential NSTEMI [3]. As a level B recommendation, these guidelines support the use of “a single negative CK-MB mass, Troponin I, or Troponin T measured 8 to 12 hours after symptom onset” to exclude the diagnosis of NSTEMI as defined by the World Health Organization [8]. These are the first emergency medicine guidelines to formally link the duration from symptom onset to the interpretation and timing of serum markers. The subcommittee further suggested that the reference range for serum markers be lowered to the 99th percentile for the normal healthy population. For Troponin I and T, this change would represent a near10-fold decrease in the threshold for considering acute coronary syndrome. The effect of this recommendation has been to increase serum marker sensitivity while raising the dilemma of how to clinically interpret results that fall within the abnormal range but below the traditional diagnostic cutoff. Although we believe these recommendations accurately reflect recent clinical studies as well as the performance characteristics of newer serum markers, most ED physicians continue to employ a traditional serial biochemical marker approach.

Although the new ACEP guidelines endorse a single negative troponin I or T for the exclusion of NSTEMI when measured 8 to 12 hours after symptom onset, there are only a few trials that have directly addressed this issue. In 1997, Hamm et al. evaluated 773 consecutive patients who presented with chest pain of <12 h duration and an absence of acute ST-elevation myocardial infarction (STEMI). Among all patients with a negative troponin I, obtained at least 6 hours after the onset of symptoms, the event (death or MI at 30 days) rate was 0.3% [9]. In 2001, Limkakeng et al. attempted to prospectively use a combination of a single TnI at presentation and a Goldman risk ≤4% to identify a subset of patients that would be appropriate for early discharge from the emergency department [10]. Of the 2,322 patients enrolled, 998 had both an initial negative TnI and a Goldman risk ≤4%. A total of 49 (4.9%) patients met the composite endpoint of death, AMI, or revascularization. The authors conclude from this study that a single cardiac TnI was insufficient to exclude the diagnosis of acute MI among a selected group of low risk patients. Also, in 2001, two articles were published that attempted to validate accelerated clinical pathways to exclude the diagnosis of AMI in the ED [11, 12]. In the paper by Ng et al. one arm of the accelerated pathway was for patients with >6 hours of symptoms thought not to be cardiac in origin. For these patients, the algorithm called for a single TnI and an ECG. If the ECG was nondiagnostic and the first TnI was negative, then the patient could be discharged home without further testing. The 30-day event rate for all patients discharged home during the study was 0.2% for MI and 0.8% readmission for unstable angina. 

The first study to evaluate a single troponin in relation to symptom duration was a small prospective study of 267 patients presenting to an ED with suspected myocardial ischemia [13]. A single troponin T (TnT) was obtained at presentation in all patients. Sixty patients were ultimately diagnosed with AMI, based on traditional WHO criteria. 52 of these 60 AMI patients had initial positive TnT upon presentation to the ED, revealing an overall sensitivity of 86.7% for single initial TnT. Among the 8 patients with initially negative TnT, the duration of symptoms was always less than 3.5 h. Therefore, in this small study, the sensitivity of an initial TnT to diagnose myocardial infarction was 100% in the subset of patients with greater than 3.5 hours of symptoms.

Although providers may be wary to discharge patients home after a single troponin I, there has been at least one retrospective chart review that looked at the results of 588 patients who were discharged home from the ED after a single normal cTnI was obtained 6–9 hours after symptom onset [14]. In this population, an adverse cardiac event (cardiac death or MI at 30 days) occurred in 2 patients (0.34%), both return visits for an NSTEMI. The authors point out that in both of these patients, the evaluation deviated from their group's standard practice. The first patient was discharged from the ED even though the troponin sample was drawn 30 minutes after the onset of symptoms, and the second patient was discharged home despite new ST segment depressions on the ECG in the setting of presumed stable angina. Neither patient died within 30 days.

The appropriate diagnostic cutoff to use for a single *or* serial marker approach, has been the subject of intense debate. The original WHO guidelines for the diagnosis of acute myocardial infarction use a cutoff for cardiac enzymes defined as “twice the upper limit of normal” [8]. Cardiac enzyme bioassays have matured substantially since this time. The European Society of Cardiology (ESC) and the American College of Cardiology (ACC) convened a conference in 2007 to readdress the issue of a standardized definition for the diagnosis of AMI [15]. This committee defined an increased cardiac troponin as “a measurement exceeding the 99th percentile of a reference control group”, meaning that 1% of the normal population will routinely fall within the abnormal range. They further define an acceptable level of imprecision, or coefficient of variance (CV), at the 99th percentile as ≤10%. Finally, the new ESC/ACC guidelines provide criteria for acute, evolving, or recent MI that require a “typical rise and gradual fall” with either ischemic symptoms, ECG changes, or a coronary artery intervention. They do not, however, provide a cutoff value for the diagnosis of MI other than the 99th percentile definition previously described. Nevertheless, most studies of acute MI continue to use a cutoff value that is defined by the receiver operating curve (ROC) that optimizes the sensitivity and specificity for the exclusion of AMI, often based upon older CK- MB standards. In some instances, the new reference value recommended by the ESC/ACC is an order of magnitude smaller than the previously recognized definition for determining myocardial ischemia. It is estimated that routine application of the new reference cutoffs will increase the diagnosis of AMI by 20%–30%. 

In our study, we identified 115 patients with an initial negative, but subsequent positive, TnI out of population of 4510 ED patients undergoing serial cTnI testing. Within this group, 25 patients (21.7%) presented with >8 h of symptoms. At first glance, this percentage seems to be unacceptably high to exclude myocardial infarction on the basis of initial troponin and symptom duration. However, one must take a closer look at the details of these 25 patients (Table 4) prior to drawing any conclusions. 

First, the new ECS-ACC recommended cutoff for the diagnosis of cardiac injury would have us use a much lower value of troponin than the ROC cutoff for AMI (i.e., 0.06 ng/mL instead of the current 0.6 ng/mL). 18 of our 25 patients who presented to the ED >8 hours after symptom onset had an initial TnI that was >0.06 ng/mL. In other words, those 18 patients had biochemical evidence of cardiac injury at the time of arrival, requiring at least one additional TnI level to further define their disease process. Many of these patients had nonischemic etiologies responsible for TnI elevation (Table 5) [15].

Of the remaining 7 patients with no biochemical evidence of cardiac injury on the initial troponin, two had a final primary diagnosis of ACS. The first was a 75-year-old woman with a history of diabetes, hypertension, and prior MI, who presented with a week of worsening chest pain, much worse “for one day” who presented with 10/10 pain. Her duration of symptoms was recorded as 24 hours, because no more specific mention of an onset was made. The other patient with an ultimate primary diagnosis of ACS was an 82-year-old female with a history of hypertension and heart failure who presented with 3 days of worsening shortness of breath. She had an LBBB on her initial ECG and an elevated initial myoglobin value. Both of these patients were high-risk patients who presented in extremis—neither of which would have been “occult” NSTEMIs missed by the new guidelines. It should be further noted that of these 7 patients, 5 of them were >60 years old with at least one other cardiac risk factor and the other 2 had recently used cocaine—risk factors or practices that would have declared them as high-risk patients who would typically be either admitted or at least subjected to serial serum cardiac testing to exclude AMI.

## 5. Limitations

This study has several potential limitations that are typical of a retrospective study design. Although the study population itself was identified using a laboratory database, the remainder of the information was obtained from a medical record review. Two authors did the entirety of the data abstraction, which could call in to question both the quality and the reliability of the data. In addition, the abstractors were not blinded to the objective of the study during the data abstraction. We feel that although this is a potential source of significant bias, we took steps to limit this. For example, we queried our troponin database using a broad definition of “initially negative, subsequently positive” troponin. By using the 0.6 ng/mL cutoff for normal rather than 0.06 ng/mL, we were certain to catch all possible cases that could have been missed with a single troponin. In addition, when determining the duration of symptoms, we required a specific mention to be made of a time of onset, or else we accepted the much longer time that is implicated by statements such as “one day”, even if it was clear that the patient had continuing or progressively worsening symptoms upon arrival to the ED. This maximizes the number of possible patients with >8 hours of symptoms upon arrival, so that we would not miss any potential cases.

Another potential limitation to this study is the fact that 187 patients had a first positive troponin upon arrival. Given the possibility that these patients may have been seen in the 7 days prior and discharged home after a single troponin, we queried our ED tracking system and found that 18 of these patients had been seen in the week prior. Two of these 18 patients had been discharged home after a single negative troponin.

## 6. Conclusions

The use of a negative initial TnI together with a symptom onset of ≥8 hours prior to serum marker testing defines a population at very low risk for a near-term diagnosis of ACS. Although we believe that our data considered in conjunction with all of the prior studies addressing this topic suggest, it is safe to exclude MI on the basis of symptom duration and initial TnI, and further research is needed to better define the population to which this strategy can be applied. The next step will be a prospective observational study assessing the ability of the combination of duration of symptoms, single TnI with the new lower cutoff, and ECG to exclude the diagnosis of acute myocardial infarction. Patients will be subclassified using the new grading system for myocardial infarction whenever possible [16].

## Figures and Tables

**Table 1:** Characteristics of patients in the study group.

Age, y (%)	*N* = 115
20–29	4 (3.5)
30–39	5 (4.3)
40–49	17 (14.8)
50–59	22 (19.1)
60–69	25 (21.7)
70–79	20 (17.4)
80–89	17 (14.8)
90–99	5 (4.3)

Gender (%)	
Male	62 (53.9)
Female	53 (46.1)

Race (%)	
African American	55 (47.8)
Hispanic	26 (22.6)
Caucasian	18 (15.7)
Asian	9 (7.8)
Other	7 (6.1)

Past Medical History (%)	
Hypertension	81 (70.4)
Diabetes	41 (35.7)
Prior myocardial infarction	41 (35.7)
Hyperlipidemia	33 (28.7)
Congestive heart failure	30 (26.1)
Prior stroke	16 (13.9)
Prior cardiac catheterization	12 (10.4)
Chronic kidney disease	9 (7.8)
Prior CABG	6 (5.2)
Family history of early myocardial infarction	6 (5.2)

Substance abuse (%)	
Tobacco	38 (33.0)
Cocaine	19 (16.5)
Alcohol	14 (12.2)
Injection drug use	1 (0.9)

CABG: coronary artery bypass graft.

**Table 2:** Presenting characteristics of the study population.

Arrival (%)	*N* = 115
Medical code	47 (40.9)
Trauma activation	9 (7.8)

Presenting symptom (%)	
Chest pain	50 (43.5)
Shortness of breath	35 (30.4)
Altered mental status	10 (8.7)
Cardiac/respiratory arrest	8 (7.0)
Syncope	4 (3.5)
Other	12 (10.4)

Duration of symptoms, h (%)	
≤1 hr	61 (53.0)
>1 hr–≤4 hr	22 (19.1)
>4 hr–≤8 hr	7 (6.1)
>8 hr–24 hr	10 (8.7)
>24 hr	15 (13.0)

ECG diagnostic of ischemia (%)	
Yes	38 (33.0)
No	77 (67.0)

Ongoing symptoms (%)	
Yes	109 (94.8)
No	6 (5.2)

ACLS (%)	
Cardioversion	8 (7.0)
Chest compression	8 (7.0)
Code drugs	9 (7.8)

ACLS: advanced cardiac life support.

**Table 3:** Outcomes of the study population.

Workup/treatment (%)	*N* = 115
Cardiac Catheterization	21 (18.3)
Stress test	17 (14.8)
Thrombolysis	12 (10.4)

Primary diagnosis (%)	
NSTEMI	39 (33.9)
STEMI	16 (13.9)
CHF exacerbation	9 (7.8)
Arrest	8 (7.0)
Tachydysrhythmia	5 (4.3)
Pulmonary embolism	5 (4.3)
Pneumonia	3 (2.6)
Pulmonary edema	3 (2.6)
Intracranial hemorrhage	3 (2.6)
Sepsis	3 (2.6)
Gastrointestinal bleed	3 (2.6)
Syncope	3 (2.6)
Hypertensive emergency	2 (1.7)
Stroke	2 (1.7)
Other	9 (7.8)

Death (%)	
Yes	14 (12.2)

STEMI: ST segment elevation myocardial infarction, NSTEMI: non-ST segment elevation myocardial infarction, CHF: congestive heart failure.

**Table 4:** Selected details of 25 patients with an initial troponin value <0.6 ng/mL, any subsequent troponin value ≥0.6 ng/mL, and ≥8 hours of symptoms.

Age gender	Chief complaint	Duration of symptoms	Initial troponin ng/mL	Peak troponin ng/mL	ECG diagnostic	Diagnosis primary, secondary
61 F	SOB	2 weeks	<0.06	0.74	No	Asthma exacerbation
69 M	SOB	3 days	<0.06	0.9	No	Pleural effusion
75 F	Chest Pain	1 day	<0.06	6.49	No	ACS-NSTEMI, pneumonia
49 M	Rib Pain	2 weeks	<0.06	1.92	No	Pneumonia, cocaine abuse, and atrial Fibrillation
22 M	Abdominal Pain	1 week	0.06	0.79	No	Hypertensive emergency, renal failure, and cocaine abuse
82 F	SOB	3 days	0.06	2.38	Yes (LBBB)	ACS-NSTEMI, CHF exacerbation
81 M	“feeling lousy”	10 hours	0.06	8.24	Yes (STD)	Bacteremia, pneumonia, and ACS-NSTEMI
73 F	SOB	1 day	0.08	0.87	No	CHF exacerbation and ESRD
70 F	Abdominal Pain	3 days	0.14	1.64	No	Acute cholecystitis
60 M	SOB	3 days	0.17	1.34	No	CHF exacerbation and Pneumonia
38 M	Chest Pain	2 days	0.17	2.32	No	ACS-NSTEMI
38 F	SOB	1 day	0.19	1.47	No	CHF exacerbation and cocaine abuse
74 M	Chest Pain	10 hours	0.22	1.24	Yes (STE)	ACS-NSTEMI
64 F	Chest Pain	12 hours	0.22	16.39	Yes (LBBB)	ACS-NSTEMI
48 M	Chest Pain	8.5 hours	0.24	3.99	No	ACS-NSTEMI
60 M	Chest Pain	4 days	0.25	3.30	No	Gastrointestinal bleed
54 M	SOB	1 day	0.31	0.89	No	Pulmonary embolism and cocaine abuse
70 F	SOB	1 week	0.31	0.7	Yes (LBBB)	CHF exacerbation
73 F	SOB	18 hours	0.35	1.13	No	Pulmonary embolism
50 M	SOB	2 weeks	0.43	0.76	No	CHF exacerbation, cocaine abuse, emphysema
75 F	Chest Pain	1 day	0.45	0.91	No	ACS-NSTEMI
67 F	Chest Pain	4 days	0.48	1.12	No	ACS-NSTEMI
64 M	Chest Pain	2 days	0.48	3.56	No	ACS-NSTEMI
49 F	Found Down	22 hours	0.56	0.94	No	Mechanical fall, closed head injury
83 F	SOB	2 days	0.58	0.66	No	Pneumonia

ACS: acute coronary syndrome, NSTEMI: non-ST segment elevation myocardial infarction, CHF: congestive heart failure.

**Table 5:** Elevations of troponin in the absence of acute myocardial ischemia.

Cardiac trauma—for example, contusion and post-surgical trauma
Congestive heart failure
Pulmonary embolism
Chronic kidney disease
Severe sepsis
Large burns with or without rhabdomyolysis
Acute stroke or subarachnoid hemorrhage
Cardiac infiltrative disease (e.g., amyloidosis, hemochromatosis, and sarcoidosis)
Cardiac inflammatory disease (e.g., myocarditis and pericarditis)
Aortic dissection of valve disease
Hypertrophic cardiomyopathy
Cardiac dysrhythmia
